# On Aneurisms

**Published:** 1896-03-28

**Authors:** A. Pearce Gould

**Affiliations:** Senior Assistant Surgeon to the Middlesex Hospital.


### On Aneurisms.

By A. Pearce Gould, F.R.C.S., Senior Assistant Surgeon to the Middlesex Hospital.

In this the concluding article on aneurism we have to notice the treatment of certain special forms and complications of that disease. The most trustworthy surgical treatment of a sacculated aneurism is the ligature of the affected artery on the proximal side of the tumour, and recent experience has shown that it is well to place the ligature near to, rather than far from, the sac. But there are cases of aneurism, especially at the root of the neck and in the mediastinum, where such an operation is impossible. In these cases the ligature of the artery on the further side of the aneurism may be employed. The results of this treatment are by no means constant, and the surgeon should advise it especially when such a ligature will arrest entirely the flow through the aneurismal artery (as in the case of aneurism of the lower part of the common carotid artery), or when he finds that digital compression of the artery to be tied greatly modifies the tension of and pulsation in the aneurism. Attempts to excite coagulation in an aneurism by more direct means, such as electrolysis and the introduction of various coagulants, are only rarely justifiable, and still more rarely successful. Of such means electrolysis appears to be the best.

Excision of an aneurism, the "old" operation, or the "operation of Antyllus," was generally abandoned, when the "Hunterian operation" was introduced; it was abandoned because of its difficulty, and its great danger from secondary hæmorrhage. For the last hundred years it has been but rarely practised, chiefly for small traumatic aneurisms in accessible situations. Of late years, since the introduction of the bloodless method of operating, and the employment of efficient antiseptics, both the difficulty and the danger of this operation have been greatly diminished, and it is likely that in the future it will be more often and more successfully practised than in the pre-Hunterian period.

When an aneurism is found to be rapidly enlarging no time should be lost in ligaturing the artery above it, and if, while enlarging fast, its outline is becoming less distinct and the impulse in it less forcible, the need for hurry is still greater. For in this last case the sac is leaking, a free rupture is likely to occur, and if it does amputation will probably be the only possible resource. When subcutaneous rupture of an aneurism has occurred, the widespread escape of blood quickly causes gangrene from the entire arrest of circulation in the part beyond. If gangrene has declared itself, the only course open to the surgeon is to amputate well above the aneurism and the level of the gangrene. But if the surgeon sees the case before the onset of definite gangrene, an effort may be made to excise the aneurism and remove as much as possible of the extravasated blood. Such an operation should only be undertaken when the surgeon knows the exact position of the aneurism and, approximately, its size and condition before it ruptured. Great care must be taken to prevent all further loss of blood during the operation; the incision in the skin should be free, so planned as readily to expose the sac without injury to other important structures, especially vessels. If this operation fail, the surgeon should at once amputates the member.

Gangrene either "dry" or "moist" may follow the ligature of an artery for aneurism. When it is of the "dry" variety, it is the result of failure of the arterial circulation, either from a weak heart or insufficient anastomotic channels or from advanced disease of the arteries exciting coagulation of the blood as it flows through them in a languid stream. Whatever its cause, it is usually limited in extent, and the tissues adjacent to the dead part are themselves only just able to maintain their vitality The dry mummified tissues do not decompose, and very little if any absorption of poisonous products occurs from them. The surgeon is, therefore, under no obligation to interfere at once, and the just-living tissues are wholly unable to withstand the depressing influence of a trauma. In such a case the best practice to follow is to wrap up the part in a porous, absorbent antiseptic dressing, to favour its complete mummification and its more thorough disinfection. Nature will speedily mark off very accurately the living from the dead tissue. When this separation is well advanced, the surgeon should amputate the limb either by completing the line of ulceration or by dividing the living tissues in a convenient manner close to the dead portion, so as not to sacrifice more than is absolutely necessary. By this time the parts adjacent to the gangrenous area will have recovered enough to enable them to bear and to repair the wound.

When the gangrene is moist, it is caused by interference with the venous return from the part. This may be due to compression of the main vein either by the aneurism or the extravasated blood or by an accident at the operation. However it arises it usually extends up to the level of the ligature. The products of decomposition of the wet gangrenous tissue are absorbed, and are very toxic. Hence it follows that the surgeon should at once, without any delay, amputate the limb above the aneurism.

Recurrence of pulsation in an aneurism after ligature of the main artery is another accident full of anxiety for the surgeon. Several alternative plans of treatment present themselves. Probably the wisest course to adopt is to place the patient at rest in bed, with the limb well raised; a bandage should then be applied all the way up the limb, a pad being placed over the aneurism, so that the pressure will be more at this point than elsewhere. Should rest and this direct pressure fail after a patient trial, the artery should be ligatured on the distal side of the previous ligature, or if the case is otherwise suitable for it, the aneurism may be excised, a certain way of ridding the patient of his trouble.

